# Detection of Pneumonia from Chest X-ray Images Utilizing MobileNet Model

**DOI:** 10.3390/healthcare11111561

**Published:** 2023-05-26

**Authors:** Mana Saleh Al Reshan, Kanwarpartap Singh Gill, Vatsala Anand, Sheifali Gupta, Hani Alshahrani, Adel Sulaiman, Asadullah Shaikh

**Affiliations:** 1 Department of Information Systems, College of Computer Science and Information Systems, Najran University, Najran 61441, Saudi Arabia; msalreshan@nu.edu.sa (M.S.A.R.); asshaikh@nu.edu.sa (A.S.); 2 Chitkara University Institute of Engineering and Technology, Chitkara University, Punjab 140401, India; kanwarpartap.gill@chitkara.edu.in (K.S.G.); vatsala.anand@chitkara.edu.in (V.A.); sheifali.gupta@chitkara.edu.in (S.G.); 3 Department of Computer Science, College of Computer Science and Information Systems, Najran University, Najran 61441, Saudi Arabia; aaalsulaiman@nu.edu.sa

**Keywords:** deep learning, classification, pneumonia, transfer learning, disease, chest X-ray images

## Abstract

Pneumonia has been directly responsible for a huge number of deaths all across the globe. Pneumonia shares visual features with other respiratory diseases, such as tuberculosis, which can make it difficult to distinguish between them. Moreover, there is significant variability in the way chest X-ray images are acquired and processed, which can impact the quality and consistency of the images. This can make it challenging to develop robust algorithms that can accurately identify pneumonia in all types of images. Hence, there is a need to develop robust, data-driven algorithms that are trained on large, high-quality datasets and validated using a range of imaging techniques and expert radiological analysis. In this research, a deep-learning-based model is demonstrated for differentiating between normal and severe cases of pneumonia. This complete proposed system has a total of eight pre-trained models, namely, ResNet50, ResNet152V2, DenseNet121, DenseNet201, Xception, VGG16, EfficientNet, and MobileNet. These eight pre-trained models were simulated on two datasets having 5856 images and 112,120 images of chest X-rays. The best accuracy is obtained on the MobileNet model with values of 94.23% and 93.75% on two different datasets. Key hyperparameters including batch sizes, number of epochs, and different optimizers have all been considered during comparative interpretation of these models to determine the most appropriate model.

## 1. Introduction

Pneumonia is a respiratory disease that causes inflammation in one or both lungs, resulting in symptoms such as cough, fever, and difficulty breathing. Early detection of pneumonia is essential for effective treatment and improved patient outcomes. Unfortunately, pneumonia is just one of several lung diseases, thus radiographic results do not always confirm a pneumonia diagnosis. Therefore, with current technology, it is impossible to distinguish pneumonia from other lung diseases with certainty using radiological criteria [1].

Developing accurate pneumonia detection algorithms requires large amounts of high-quality labeled data, which can be difficult to obtain. This is particularly challenging in the case of pneumonia, where expert radiologists are required to label the data, and the number of available labeled images is limited. Deep learning, a subset of artificial intelligence, has emerged as a powerful tool for detecting and diagnosing pneumonia from medical images such as chest X-rays [2].

Deep-learning algorithms can be trained on large datasets of chest X-rays to recognize patterns and features that are indicative of pneumonia. This involves using convolutional neural networks (CNNs), a type of deep-learning architecture that is particularly well suited to image recognition tasks. By analyzing the texture, shape, and intensity of pixels in chest X-rays, CNNs can learn to identify regions of the image that correspond to areas of infection or inflammation in the lungs [3].

Once trained, deep-learning models can be used to classify new chest X-rays as either showing signs of pneumonia or not. This can be done in real time, making it a potentially valuable tool for healthcare professionals in diagnosing and treating patients with pneumonia. Additionally, deep-learning models can be used to assist radiologists in interpreting chest X-rays, reducing the risk of misdiagnosis and improving patient outcomes [4].

The following list summarizes the main contributions of the research that is being proposed:

- In this research, a MobileNet model has been proposed to detect pneumonia. The model is simulated on two datasets having 5856 and 112,120 chest X-ray images.
- The performance of the proposed MobileNet model has been compared with ResNet50, ResNet152V2, DenseNet201, EfficientNet, Xception, VGG16, and DenseNet121 in terms of accuracy, precision, recall, F1-score, and the area under the curve (AUC).
- The proposed model has been simulated with different optimizers namely ADAM, ADADELTA, and SGD with different batch sizes and epochs of 16, 32, and 64.

The other parts of the paper are as follows, Section 2 shows related work, Section 3 comprises the proposed methodology, the results and discussion are explained in Section 4, and the conclusion is shown in Section 5.

## 2. Related Work

In recent years, a number of techniques, especially a few profound deep-learning strategies, have been proposed to briefly layout a strategy in pneumonia diagnosis utilizing chest X-ray images. The authors [5] had worked on ResNet18 using 349 chest X-ray images and were performing classification using X-ray images on two classes, namely pneumonia and non-pneumonia, showing an accuracy of 99.4%; however, the number of images was very small. The authors in [6] had worked on CoviWavNet using 11,164 CT scans and were performing classification on two classes, namely SARS-CoV and normal, showing an accuracy of 99.33%. The authors in [7] worked on the VGG16 model using 12,146 CT scans and performed classification on three classes, namely COVID-19, pneumonia, tuberculosis, and healthy cases showing an accuracy of 99.12%. The authors in [8] had worked on ResNeT101 using 2482 chest X-ray images and performed classification on two classes, namely COVID-19 and non-COVID-19, showing an accuracy of 99%. The authors in [9] had worked on VGG16 using 7000 chest X-ray images and performed classification on three classes, namely novel coronavirus pneumonia, patients with common pneumonia (CP), and normal controls showing an accuracy of 93.57%

Ksibi et al. used a pre-trained ResNet model on ImageNet weights. The maximum degree of accuracy was 98.34%, which was higher than the accuracy attained by other cutting-edge techniques analyzed in past studies [10]. Luz et al. had displayed a tall execution within the classic ImageNet dataset whereas showing it as a little division would have taken a toll on other popular architectures such as the ResNet and VGGs. This proposed model achieves a high accuracy value of 93.9% [11]. The dataset, which included 112,120 chest X-ray pictures from 30,805 patients was used by Rajpurkar et al. There are training and test sets for the full dataset. The photos are scaled down to 224 × 224 and normalized using metrics from the ImageNet16 training dataset. These photos are used to train the CheXNet model, which uses the DenseNet 121 layered Dense CNN. The classification job was pneumonia/no pneumonia; hence, this layer was changed to a single sigmoid neuron. The classes pneumonia and non-pneumonia (14 classes including other lung disorders) were quite unbalanced because the NIH dataset has 15 classes. When tested with 420 photos, the model finished with an F1-score of 0.435 and an AUROC of 0.76 [12]. Pak KinWonga et al. recognized that COVID-19 pneumonia, non-COVID-19 viral pneumonia, bacterial pneumonia, mycoplasma pneumonia, and typical lung on chest CT images play a pivotal part in opportune isolation and restorative treatment. The test appears to be that the proposed MSANet can accomplish an overall precision of 97.31%, a value of recall of 96.18%, a value of F1-score of 96.71%, a value of accuracy of 97.46%, and a macro-average region beneath the recipient working characteristic bend (AUC) of 0.9981 to recognize between numerous classes of pneumonia [13]. Furtado et al. used Cimatec_XCOV19, a novel deep-learning system inspired by the Inception-V3 architecture that can support the identification of abnormal chest radiographs and classify the abnormal radiographs as suggestive of COVID-19. The Cimatec_XCOV19 algorithm obtained a sensitivity value of 0.85, a specificity value of 0.82, and an AUC ROC of 0.93. The AUC ROC of the algorithm is compared with a well-known public solution and did not find a statistically relevant difference between their performances [14]. Cohen GP and Kagel et al. utilized the combination of two pictures from an open-source dataset collected by them. The information comprises four categories, namely normal pneumonia, bacterial pneumonia, viral pneumonia, and COVID-19, for 2433 images. It was considered for Xception and ResNet50 [15]. Barhoom et al. utilized deep-learning models for pneumonia classification by giving whole X-ray images to extricate and learn one of kind of X-ray image from both ordinary and pneumonia classes within the dataset. The diverse deep-learning models utilized in comparing and recognizing pneumonia were CNN_1, CNN_2, DenseNet121, VGG16, ResNet50, and InceptionV3 [16]. Mahmoudi et al. created a conclusion framework based on profound learning methods to identify and measure COVID-19 contamination and pneumonia screening utilizing CT imaging. A U-net design, based on CNN encoder and CNN decoder approaches, was at that point presented for a quick and exact picture to get lung and contamination segmentation models. The test illustrated that the proposed framework accomplishes a dice score of 0.98 and 0.91 for lung and contamination division errands individually and a precision of 0.98 for classification assignment [17]. Chhabra, M et al. have proposed an effective ResNet-50 exchange learning-based convolutional neural arrangement to anticipate pneumonia utilizing restorative pictures. A Kaggle-based open-source dataset store is utilized for the test investigation [18]. In the proposed work, MobileNet architecture is used for the classification of pneumonia and non-pneumonia classes with 5863 chest X-ray images. Very little work has been implemented on the classification of pneumonia and non-pneumonia classes with chest X-ray images. Necessary changes have been implemented in the updated manuscript. Here, in the proposed work, MobileNet architecture is used for the classification of pneumonia and non-pneumonia classes with 5863 and 112,210 chest X-ray images.

## 3. Proposed Methodology

**Figure 1:**
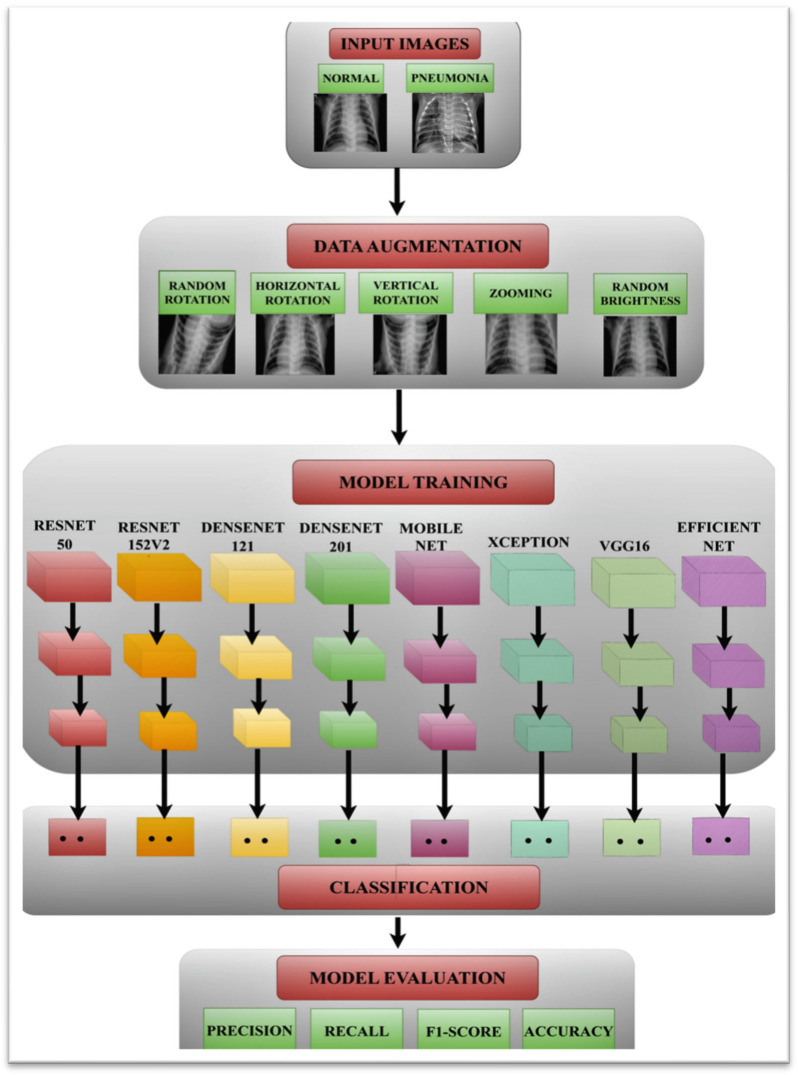
Proposed methodology.

The proposed methodology employs a number of stages, which are covered in more detail in the following sections, to diagnose pneumonia. Figure 1 depicts the suggested methodology for an automated diagnosis of pneumonia. This model’s objective is to categorize chest X-ray pictures into normal and pneumonia classes. The original chest X-ray pictures are used as a base for the data augmentation procedures. The pre-trained models are used in conjunction with the augmented images to classify pneumonia. The following sections go into great detail about each level.

### 3.1. Input Dataset

**Figure 2:**
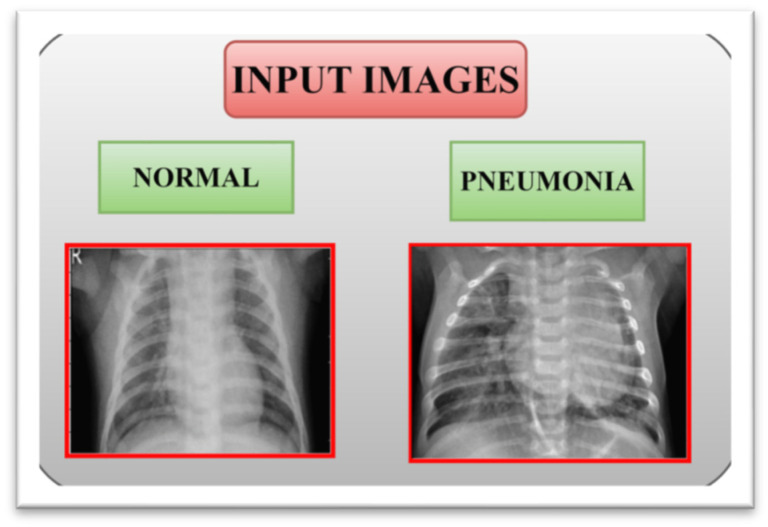
Sample chest X-ray image of normal and pneumonia.

Here, the pneumonia-chest X-ray dataset is utilized to gather pneumonia X-ray pictures that consider images from different open sources and which has been overhauled routinely. Here, two datasets are used to train the models for diagnosing pneumonia. The first dataset consists of 5856 images of chest X-rays of which 4273 are pneumonia images and 1583 are normal chest X-ray images [19]. A total of 80% of the data are used for training, producing 4642 images (3418 images of pneumonia and 1224 normal images), 15% of the data are used for testing, producing 919 images (641 cases of pneumonia and 278 normal images), and the final 5% of the data are used for validation (214 cases of pneumonia and 81 non-pneumonia images). Figure 2 shows the chest X-ray sample images of normal and pneumonia classes.

The second dataset taken is ChestX-ray14 which contains 112,120 chest X-ray images of 30,085 individuals. Out of these 112,120 images, 1431 images had pneumonia labels on them. To take a balanced dataset, 1431 normal X-ray images (labeled with ‘No Findings’) were chosen from the dataset. Hence the finally taken dataset has 1431 pneumonia images and 1431 normal X-ray images. 80% of the data are used for training, producing 2290 images (1145 images each of pneumonia and normal images), 5% of the data are used for validation, producing 142 images (215 images each of pneumonia and normal images), and the final 15% of the data are used for testing, producing 430 (71 images of pneumonia and normal images).

**Figure 3:**
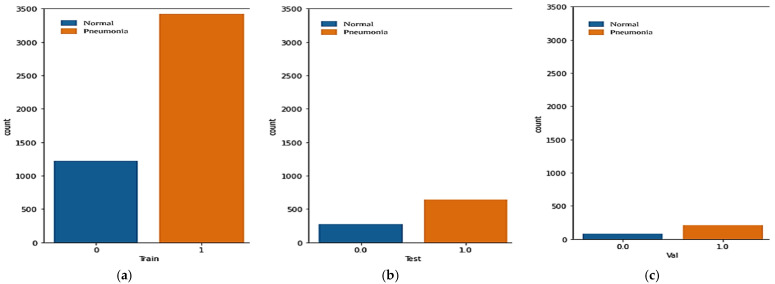
Count plot depicting count for the pneumonia and normal dataset: (**a**) training set (**b**) testing set (**c**) validation set.

The count plot of the first dataset is used to depict counts for pneumonia and normal images. In order to display all images, Figure 3 shows that the training set’s x-axis contains values of 0 (which represents 1224 normal images) and 1 (which represents 3418 pneumonia images), while the testing set’s x-axis contains values of 0 (which represents 278 normal images) and 1 (which represents 641 pneumonia images), and the training set’s y-axis displays the count plot of both pneumonia and normal images.

The training, testing, and validation datasets’ count plot is depicted to convey the proper format of the images used in the dataset for model prediction. There are three classes under which the original dataset is reviewed, i.e., pneumonia, normal, and total images and then the representation is further done under three parameters, i.e., training, validation, and testing.

The details of data splitting on the training, validation, and testing classes of data are evaluated in Table 1 and the evaluation of various CNN models will be done on this basis.

### 3.2. Data Augmentation

**Figure 4:**
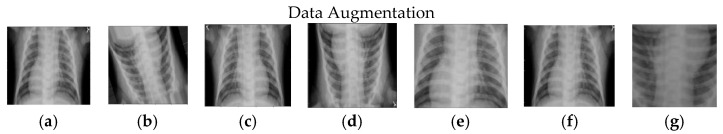
Data augmentation of dataset images for enhancement: (**a**) original image; (**b**) random rotation; (**c**) horizontal rotation; (**d**) vertical rotation; (**e**) zoom; (**f**) random brightness; (**g**) resize.

The CNN models need a large number of data sources for optimal training to demonstrate improved performance on larger datasets. Since there is only a small dataset being used, this is employed to artificially enhance the dataset. This also aids in avoiding overfitting. The approach of data augmentation has been frequently used and increases the number of pictures by applying a series of changes while maintaining class labels. Data augmentation is applied to training images of the pneumonia class to increase the images’ diversity, which also acts as a dataset regulator. Data augmentation has also been applied to the non-pneumonia (normal) images of the first dataset to increase its image number from 1224 to 3672 to balance the dataset. The number of training images of the pneumonia classes total 3418 whereas the number for the non-pneumonia class is 3672. No augmentation is performed on testing images and validation images of the dataset. Figure 4 illustrates the methods used in this work to enhance the training images. The methods that are used to enhance the images include random rotation, horizontal flipping, vertical rotation, zooming, random brightness, and resizing of the images.

### 3.3. Pneumonia Prediction Using Pre-Trained Models

When a large dataset is typically trained on a wide-scale image classification task then that saved network is termed a pre-trained model. The model can be customized while using either a pre-trained model or transfer learning according to the data augmentation task, as shown in Figure 4.

For transfer learning, the concept is that, in case a demonstration is prepared on a huge and general dataset, then, at that point, the image classification can be done with the assistance of the highlight maps without having to hustle from scratch by preparing a huge demonstration on a huge dataset.

Figure 1 shows all the pre-trained models used in this study. One of the most potent deep neural networks, ResNet, excelled in the 2015 ILSVRC classification challenge, achieving fantastic performance outcomes. ResNet also demonstrated strong generalization performance on other identification tasks. The ResNet architecture has various variations, all of which use the same basic idea but a different number of layers. In this paper, ResNet50 and ResNet152V2 models are used. Every ResNet plan employs 77 and 33 kernel sizes for the introductory convolution and max pooling, separately. The diagram below illustrates the ResNet50 architecture, which consists of four stages. The assumed input size is 224 × 224 × 3 for an explanation. In ResNet50 and ResNet152v2, a total of three layers are piled on top of one another for each residual function. Convolutions (one, three, and one) make up the three levels. The reduction and subsequent restoration of the dimensions are accomplished by the 11 convolution layers. With lower input and output dimensions, the 33 layers are left as a bottleneck.

Table 2 also offered information on each of the five pre-trained models [20,21]. In DenseNet, the input image is convoluted numerous times to provide high-level features. Each layer in DenseNet receives additional information from all levels that came before it and transmits its maps to all layers that came after it. Concatenation is employed. Each layer is receiving “collective knowledge” from the levels that came before it. It uses two types of DenseNet models. These are DenseNet121 and DenseNet201. Apart from the fundamental convolutional and pooling layers, DenseNet is made up of two significant building elements. They consist of Transition layers and Dense Blocks. A basic convolution and pooling layer forms the foundation of DenseNet. The MobileNet model utilized profoundly shrewd distinct convolution layers. When compared to a network within the nets, the number of parameters is dramatically diminished. Lightweight deep neural networks are delivered as a result of this. Table 2 describes the layers, parameters (in millions), input layer size, and output layer size that make up pre-trained models.

In Table 2, the MobileNet model includes 28 layers, counting depth-wise and point-wise solutions as separate layers. It has 13 million parameters with 3 million for the body and 10 million for the top layer, as is customary, it has an input layer size of 224 × 224 × 3. In addition, the other pre-trained model is ResNet50, which is 50 layers deep. It trained the network with 25.6 million images. ResNet152V2 has 164 layers which are used in training 60.4 million parameters. DenseNet has two models, DenseNet 201 and DenseNet 121, which are used to train the network on 201 and 121 layers with 20.2 and 8.1 million of parameters, respectively.

This paper uses eight pre-trained models, namely MobileNet, ResNet50, ResNet152V2, DenseNet201, DenseNet121, Xception, VGG16, and EfficientNet. Figure 1 displays the block diagrams of these deep CNN models that had already undergone training.

### 3.4. Performance Parameters

The performance of the model will be assessed utilizing the parameter named accuracy, which calculates the percentage of accurate predictions made by the model, and is examined as one of the most important metrics. The formula for accuracy is shown in Equation (1).

A = T_P + T_N/(T_P + T_N + F_P + F_N) (1)

Precision (P) is the ratio of true positives (T_P), i.e., correct predictions to the total number of relevant findings, which is the sum of true positives and false positives (F_P). The formula for ‘P’ is shown in Equation (2).

P = T_P/(T_P + F_P) (2)

Recall (R) is the ratio of T_P to the total sum of T_P and false negatives (F_N). The recall formula is shown in Equation (3).

R = T_P/(T_P + F_N) (3)

The harmonic mean of ‘P’ and ‘R’ is called the F1-score (F1). The formula for the F1-score is shown in Equation (4).

F1 = 2 × (T_P × F_P)/(T_P + F_P)(4)

The AUC score is a graphical measure used to assess the effectiveness of a binary classification model. When assessing machine learning tasks, the AUC score is frequently employed.

## 4. Results and Discussion

Here, eight pre-trained models are trained and simulated on the two datasets containing 5856 and 112,120 chest X-ray images. Out of these 112,120 images, 1431 images had pneumonia labels on them. To take a balanced dataset, 1431 normal X-ray images (labeled with ‘No Findings’) were chosen from the dataset. The performance of each model is depicted in terms of confusion matrix parameters. The representation of the training performances of different CNN models using hyperparameters such as epochs, loss, binary accuracy, mean absolute error (MAE), val_loss, val_binary accuracy, and val_mae is given in Table 3. Using these parameters, the prediction of the best CNN model is done.

From Table 3 it can be analyzed that, out of the eight training models, the MobileNet model has outperformed all other models in terms of loss, binary accuracy, validation loss, validation binary accuracy, MAE, and validation MAE, having values of 0.1368, 0.9479, 0.3013, 0.8935, 0.0755, and 0.1244, respectively. The models that show the second and the third highest values of accuracy are DenseNet121 and ResNet50, where the values of accuracy are 0.9421 and 0.9413, respectively.

The depiction of the training performances of different CNN models using various confusion matrix parameters is given in Table 4. Using these parameters, the prediction of the best CNN model is done.

From Table 4, we conclude that MobileNet is the best CNN model among the above eight CNN models. Whereas it can be observed that the other CNN models have lesser accuracies. Table 4 shows that the MobileNet model performed the best overall, with an accuracy rating of 90.85, a value of precision of 95.28, a value of recall of 91.41, and a value of the F1-score 91.41 when compared to other CNN models.

The paired Student’s *t*-test is performed to compare the performance of different deep-learning models used here to predict pneumonia. To compare their performance, the test and training sets are taken from the same set of instances as the dataset. The accuracy of different models is predicted at ten iterations. To perform a paired Student’s *t*-test, the difference in accuracy for every pair of models is taken to test whether the mean difference between the two paired samples is statistically significant or not. For this, two hypotheses are made. The first is the Null Hypothesis (H0) in which the mean difference between the two model’s predictions is zero, which means there is no difference in the performance of paired models. The second hypothesis is the Alternate Hypothesis (H1), in which the mean difference between the two model’s predictions is not zero, which means that there is a difference in the performance of the paired models. After that, t-statistics are calculated using the formula given in Equation (5)

t = (mean of the differences)/(standard deviation of the differences/sqrt (sample size)) (5)

Then, using statistical software, the *p*-value is calculated for the evaluated value of t and number of degrees of freedom N-1, where N is 10. During the *p*-value calculation, the significance level is taken as alpha = 0.05. The *p*-value of the MobileNet model shows a significant difference with respect to seven models, i.e., ResNet50, ResNet152V2, DenseNet201, VGG16, Xception, DenseNet121, and EfficientNet. The average *p*-value for these five models is coming out as 0.00457. Since this *p*-value is less than our significance level of 0.05, hypothesis ‘H0′ is rejected for MobileNet paired models, and we conclude that the MobileNet model works better than the other seven models.

### 4.1. Analysis of the Best Model with Different Optimizers

**Figure 5:**
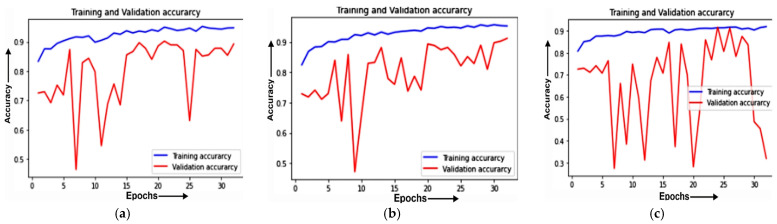
Training and validation accuracy of the MobileNet model for: (**a**) the ADAM optimizer; (**b**) ADADELTA optimizer; (**c**) SGD optimizer.

**Figure 6:**
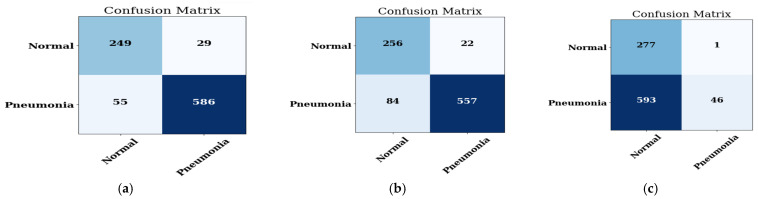
The confusion matrix of the MobileNet model on: the (**a**) ADAM optimizer; (**b**) ADADELTA optimizer; (**c**) SGD optimizer.

After the analysis of Table 4, it can be concluded that the MobileNet model has achieved a value of accuracy of 90.85%, which is the best value of accuracy as compared to the other models. This pre-trained MobileNet model is analyzed on different optimizers to calculate the accuracy, loss, and confusion matrix. Figure 5 lists the training performance in terms of training loss, validation loss, and validation accuracy for various networks using various optimizers. The confusion matrix for the same is depicted in Figure 6.

#### 4.1.1. Training and Validation Curve

The training and validation accuracies of MobileNet on different optimizers is evaluated. The Training and validation accuracy for the ADAM optimizer is depicted in Figure 5a, on ADADELTA in Figure 5b, and on SGD in Figure 5c.

Figure 5a shows the training and validation accuracy for the ADAM optimizer. It can be seen from the figure that the value of the training accuracy is constant from an epoch value of 26 to an epoch value of 30. The values of training accuracy and validation accuracy are above 90% and approximately 88%, respectively.

Figure 5b depicts the training and validation accuracy for the ADADELTA optimizer. It can be seen from the figure that the value of accuracy is increasing from an epoch value of 21 to an epoch value of 24. The value of validation accuracy shows a sudden increase from 10% to 88% and then shows a stable increase in value after that. The training accuracy’s value is above 90% whereas its value was constant from an epoch value of 15 to an epoch value of 20.

Figure 5c exhibits the training and validation accuracy for the SGD optimizer. The figure indicates that the value of validation accuracy is continuously increasing from an epoch value of 5 to an epoch value of 30. It can also be noticed that, at an 85% value, the training and validation accuracy lines meet at epoch values of 24 and 26, giving a glimpse of a steady development in accuracy.

#### 4.1.2. Confusion Matrix

The pneumonia and non-pneumonia dataset is considered for the prediction. The confusion matrix of the MobileNet model on three optimizers, namely ADAM, ADADELTA, and SGD, is illustrated in Figure 6a, on ADADELTA in Figure 6b, and on SGD in Figure 6c.

It is evaluated from the confusion matrices that when the prediction is done using the normal and pneumonia image datasets, then the MobileNet CNN model shows the best accuracy on the ADAM optimizer as compared to the other two optimizers, i.e., ADADELTA and SGD.

The representation of accuracy, precision, recall, F1-score, and AUC score of the MobileNet model on three optimizers, namely ADAM, ADADELTA, and SGD, is done and the performance of every optimizer is calculated. The performance is depicted in Table 5.

From Table 5 it is obvious that the MobileNet model obtains the best results on ADAM optimizer with a value of accuracy of 90.85, a value of precision of 95.28, a value of recall of 91.41, a value of F1-score of 91.41, and a value of AUC of 0.933 when compared to other optimizers such as ADADELTA and SGD.

### 4.2. Analysis of Best Model with Different Batch Sizes

**Figure 7:**
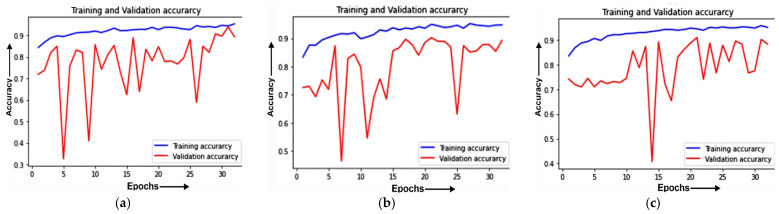
Training and validation accuracy of MobileNet model for: (**a**) 16 batch size; (**b**) 32 batch size; (**c**) 64 batch size.

**Figure 8:**
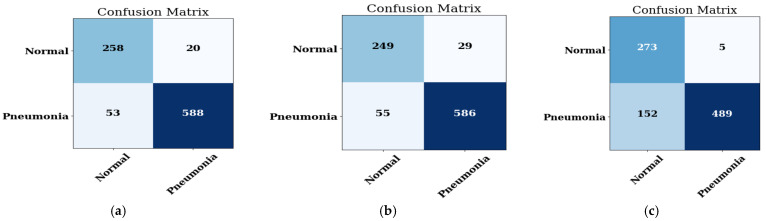
The confusion matrix of the MobileNet model on: (**a**) 16 batch size; (**b**) 32 batch size; (**c**) 64 batch size.

After the analysis from Table 5, the accuracy of MobileNet (90.85) is better than the other models and shows the best accuracy on the ADAM optimizer. This pre-trained MobileNet model is now analyzed on different batch sizes to calculate the accuracy, loss, and confusion matrix. The performance obtained by distinctive networks at distinctive batch sizes is recorded in Figure 7. The confusion matrix for the same is depicted in Figure 8.

#### 4.2.1. Training and Validation Curve

The Training and Validation accuracies and losses of MobileNet on different batch sizes are computed. The Training and validation accuracy of MobileNet on a 16-bit batch size is depicted in Figure 7a, a 32-bit batch size in Figure 7b, and a 64-bit batch size in Figure 7c.

The validation and training accuracy for the value of batch size 16 is shown in Figure 7a. The value of the training accuracy remains the same from an epoch value of 14 to an epoch value of 18. From 35% to 85% on epoch values 4–6 and 7–11 the validation accuracy has increased consistently. The values of training accuracy and validation accuracy are above 90% and approximately 88%, respectively. After the constant increase in accuracy, the training and validation lines meet at a common point at 82% on epoch value 31.

For a batch size of 32, the training and validation accuracy is depicted in Figure 7b. It can be observed from the graphical representation that the value of the validation accuracy is increasing from an epoch value of 11 to an epoch value of 24 at a progressive rate. The value of the training accuracy shows an increase of 5 points from 90% to 95% on the value of epoch increasing from 10 to 21.

Figure 7c exhibits the values of the training and validation accuracy for the batch size of 64. The figure indicates that the value of the training accuracy is continuously increasing from an epoch value of 1 to an epoch value of 30 with the increase in validation accuracy exceeding 90% and this depicts the maximum accuracy rate as compared to the training and validation accuracy on other batch sizes.

#### 4.2.2. Confusion Matrix

The pneumonia and non-pneumonia datasets are reviewed for prediction. The output is extracted in the form of confusion matrices. The matrix of MobileNet on the 16-bit batch size is depicted in Figure 8a, the 32-bit batch size in Figure 8b, and the 64-bit batch size in Figure 8c.

It is evaluated from the confusion matrices that, when the prediction is done using the normal and pneumonia image datasets, then the MobileNet CNN model shows the best performance on the 16 batch size as compared to the other two batch sizes, i.e., 32 batch size and 64 batch size.

The representation of the accuracy, precision, recall, F1-score, and AUC score of the MobileNet model on the three batch sizes, namely 16, 32, and 64, is done and the performance on every batch size is calculated. The performance is depicted in Table 6.

When compared to other batch sizes like 32 and 64, Table 6 shows that the MobileNet model performed best on the batch size 16, with values of accuracy of 92.05, precision (%) of 96.71, recall of 91.73, F1-score of 94.15, and AUC of 0.980.

### 4.3. Analysis of Best Model with Different Epochs

**Figure 9:**
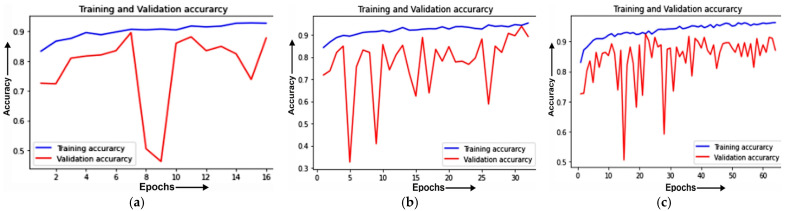
Training and validation accuracy of MobileNet model (**a**) 16 epoch; (**b**) 32 epoch; (**c**) 64 epoch.

**Figure 10:**
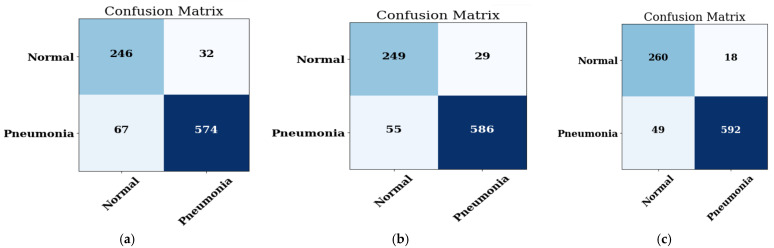
The confusion matrix of the MobileNet model on: (**a**) 16 epoch; (**b**) 32 epoch; (**c**) 64 epoch.

After the analysis from Table 5 and Table 6, the accuracy of MobileNet is best compared to other models and shows the best accuracy on the ADAM optimizer with a 16 batch size. This pre-trained MobileNet model is now analyzed on different epochs to calculate the accuracy, loss, and confusion matrix. Figure 9 lists the training performance for various networks at various epochs in terms of training loss, validation loss, and validation accuracy. Figure 10 shows the confusion matrix for the same.

#### 4.3.1. Training and Validation Curve

The Training and validation accuracies and losses of MobileNet on different epochs are projected. The Training and validation accuracy of MobileNet on 16 epochs is depicted in Figure 9a, 32 epochs in Figure 9b, and on 64 epochs in Figure 9c.

Figure 9a shows the validation and training accuracy for the value of epoch 16. The training accuracy is increasing from the epoch of value 1 until the epoch of value 12 and after that the increase in the curve is constant. The nearest point where the training and validation accuracy curves meet is 85%. The percentage values of the training accuracy and validation accuracy are above 90% and approximately 88%, respectively. After the constant increase in accuracy, the validation accuracy becomes stable at the value of epoch 10 where the accuracy percentage is 82%.

The validation accuracy and training of 32 epoch values are depicted in Figure 9b. The training accuracy curve is moving at a constant pace and the validation accuracy curve has an increase in value with an increase in the epoch values. The point where both the training and validation curves meet is at 85% on epoch value 30.

Demonstration of the training and validation accuracy for epoch 64 is depicted in Figure 9c. The figure shows that there is an inconsistent increase in the validation accuracy on various epoch values such as epoch value 15 and epoch value 30 but, after some epochs, the rate of elevation to which the validation accuracy curve goes is similar to the training accuracy curve. The values of training and validation accuracies are above 90% and 89%, respectively.

#### 4.3.2. Confusion Matrix

The pneumonia and non-pneumonia datasets are calibrated for an indication of output. The yield is extricated in the shape of confusion matrices. The MobileNet confusion matrix on 16 epochs is depicted in Figure 10a, 32-bit epoch in Figure 10b, and 64-bit epoch in Figure 10c.

It is determined from the confusion matrices that, when the prediction is done using the normal and pneumonia image datasets, then the MobileNet CNN model shows the best performance on 64 epochs when compared to other epochs, i.e., 16 epochs and 32 epochs.

The representation of accuracy, precision, recall, F1-score, and AUC score of the MobileNet model on three epochs, namely 16, 32, and 64, is done and the performance on every epoch is calculated. The performance is depicted in Table 7.

**Figure 11:**
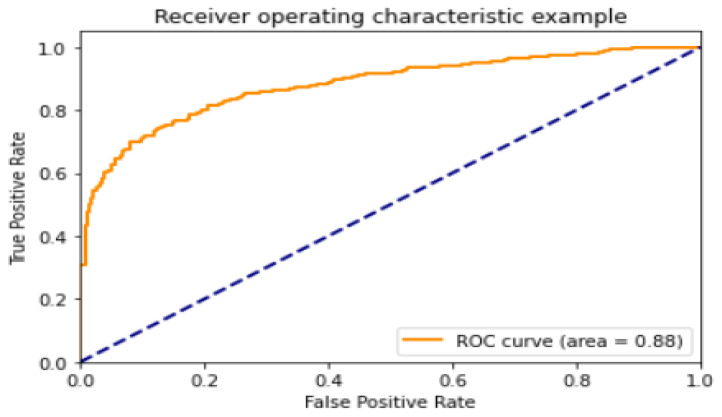
The ROC curve for the best MobileNet model.

Table 7 shows that, in contrast to other epochs, such as 16 and 64, the MobileNet model stood out on 64 epochs with values of accuracy of 92.05, precision of 96.71, recall of 91.73, F1-score of 94.15, and AUC of 0.980 and the ROC curve depicts a value of 0.88, as shown in Figure 11.

#### 4.3.3. Evaluation of Best Model at Different Datasets

From the last sections, it can be analyzed that the MobileNet model works best with the ADAM optimizer with 64 epochs and 16 batch sizes on the first dataset. Therefore, the MobileNet model is simulated on another dataset with the same hyperparameters, i.e., ADAM optimizer, 64 epochs, and 16 batch sizes.

The second dataset taken is ChestX-ray14, which was published by Wang et al. [22] and contains 112,120 chest X-ray images of 30,085 individuals. Out of these 112,120 images, 1431 images had pneumonia labels on them. To take a balanced dataset, 1431 normal X-ray images (labeled with ‘No Findings’) were chosen from the dataset. Hence, the finally taken dataset has 1431 pneumonia images and 1431 normal X-ray images. Further, this dataset was simulated with the MobileNet model with the same hyperparameters as discussed above. The results for both datasets are shown in Table 8.

#### 4.3.4. State-of-Art Comparison (SOTA)

In this section, the proposed model is compared with the work of other researchers that have worked on pneumonia and COVID diagnoses using different techniques and different datasets. From Table 9, it can be analyzed that the authors in [22,23,24,25] had worked on the diagnosis of COVID. Whereas the authors in [5,9,26,27,28,29,30,31,32,33] had worked on the diagnosis of pneumonia. The authors in [22,23,25] achieved higher accuracy than the proposed model, but they worked on the diagnosis of COVID whereas the proposed model is used for diagnosing pneumonia. Moreover, the author in [5] also achieved a good accuracy of 99.4% but had worked on a much smaller number of images, i.e., 349.

## 5. Conclusions

To separate pneumonia instances from typical cases, the power of five pre-trained CNN models, namely ResNet50, ResNet152V2, DenseNet121, DenseNet201, and MobileNet, is analyzed. The best result is obtained by MobileNet on 16 batch sizes, 64 epochs, and the ADAM optimizer. The predictions have been validated on publicly available chest radiological images. The accuracy measured using the MobileNet model is 94.23. These metrics will let analysts come up with ideas for the cure of more beneficial CNN-based models for COVID-19 preliminary resolutions.

## Figures and Tables

**Table 1:** Details of data splitting.

Class	First Dataset		
	Train	Validation	Test
Pneumonia	3418	214	641
Normal	1224	81	278
Total	4642	295	919
	Second Dataset		
Pneumonia	1145	71	215
Normal	1145	71	215
Total	2290	142	430

**Table 2:** Descriptions of the pre-trained CNN models utilized in this work, including their architecture.

Model	Layers	Parameters (in Millions)	Input Layer Size	Output Layer Size
MobileNet	28	13	224 × 224 × 3	(2,1)
ResNet50	50	25.6		
ResNet152V2	164	60.4		
DenseNet201	201	20.2		
DenseNet121	121	8.1		
Xception	71	22.8		
VGG16	16	138		
EfficientNet	10	8.4		

**Table 3:** Epoch-wise assessment of eight pre-trained models on the first dataset.

Model	Epochs	Loss	Binary Accuracy	MAE	Val_Loss	Val_Binary_ Accuracy	Val_Mae
MobileNet	8	0.1996	0.9151	0.1160	0.6880	0.8289	0.2061
	.	.	.	.	.	.	.
	.	.	.	.	.	.	.
	32	0.1368	0.9479	0.0755	0.3013	0.8935	0.1244
ResNet50	8	0.1934	0.9221	0.1075	2.6490	0.4106	0.5786
	.	.	.	.	.	.	.
	.	.	.	.	.	.	.
	32	0.1465	0.9413	0.0820	34.8674	0.2890	0.7098
ResNet152V2	8	0.2059	0.9163	0.1204	1.2353	0.3270	0.5921
	.	.	.	.	.	.	.
	.	.	.	.	.	.	.
	32	0.1690	0.9336	0.0969	1.6554	0.7833	0.2140
DenseNet201	8	0.2524	0.8931	0.1488	1.2489	0.3916	0.5913
	.	.	.	.	.	.	.
	.	.	.	.	.	.	.
	32	0.1604	0.9351	0.0891	3.9718	0.3802	0.5980
DenseNet121	8	0.2195	0.9085	0.1252	2.5500	0.7148	0.2883
	.	.	.	.	.	.	.
	.	.	.	.	.	.	.
	32	0.1557	0.9421	0.0846	0.7925	0.8251	0.1894
Xception	8	0.2206	0.9108	0.1214	3.0510	0.7681	0.2259
	.	.	.	.	.	.	.
	.	.	.	.	.	.	.
	32	0.1403	0.9442	0.0780	0.5172	0.8403	0.1698
VGG-16	8	0.6946	0.7232	0.4992	0.6934	0.2776	0.5001
	.	.	.	.	.	.	.
	.	.	.	.	.	.	.
	32	0.6918	0.3599	0.5004	0.6938	0.2776	0.5003
EfficientNet	8	0.6946	0.7232	0.4992	0.6934	0.2776	0.5001
	.	.	.	.	.	.	.
	.	.	.	.	.	.	.
	16	0.6918	0.3599	0.5004	0.6938	0.2776	0.5003

**Table 4:** Comparison of eight CNN models in terms of confusion matrix parameters on the first dataset.

Model	Accuracy (%)	Precision (%)	Recall (%)	F1-Score
MobileNet	90.85	95.28	91.41	91.41
ResNet50	30.57	100	0.4680	93.10
ResNet152V2	84.65	82.38	99.21	90.02
DenseNet201	34.27	100	5.772	91.01
DenseNet121	88.90	88.33	96.87	92.41
Xception	87.59	91.75	90.32	91.03
VGG16	30.20	85.21	43.16	93.13
EfficientNet	51.02	86.21	45.85	90.10

**Table 5:** Evaluation of MobileNet model on different optimizers with confusion matrix parameters.

Optimizer	Accuracy (%)	Precision (%)	Recall (%)	F1-Score	AUC
ADAM	90.85	95.28	91.41	91.41	0.933
ADADELTA	88.46	96.20	86.89	91.31	0.971
SGD	35.14	97.87	7.176	13.37	0.867

**Table 6:** Evaluation of MobileNet model for different batch sizes on different parameters.

Batch Size	Accuracy (%)	Precision (%)	Recall (%)	F1-Score	AUC
16	92.05	96.71	91.73	94.15	0.980
32	90.85	95.28	91.41	93.31	0.970
64	82.91	98.98	76.28	86.16	0.971

**Table 7:** Evaluation of MobileNet models on different epochs on different parameters.

Epochs	Accuracy (%)	Precision (%)	Recall (%)	F1-Score	AUC
16	89.22	94.71	89.54	92.06	0.955
32	92.05	96.71	91.73	94.15	0.980
64	94.23	93.75	98.28	95.96	0.972

**Table 8:** Results of MobileNet model on different datasets.

Number of Images	Accuracy (%)	Precision (%)	Recall (%)	F1-Score (%)
5856	94.23	93.75	98.28	95.96
112,120	93.75	91.36	94.39	93.18

**Table 9:** SOTA on chest X-ray images using different techniques.

Ref/Year	Technique	Classes	Number of Images	Accuracy
Based on COVID-19 Detection				
[22]/2021	GoogleNet	Normal and novel COVID-19	5000	97.89%
[23]/2022	DC-Net-R	Normal and COVID-19	296	96.13%
[24]/2022	ResNet50v2	Covid and Non COVID	2756	87%
[25]/2022	ResNet50V2	COVID-19 and non-COVID-19	2458	97.75%
Based on Pneumonia Detection				
[5]/2021	ResNet18	Pneumonia, Non-pneumonia	349	99.4%
[9]/2021	VGG16	novel coronavirus pneumonia, patients with common pneumonia (CP), and normal controls	7000	93.57%
[26]/2021	AlexNet	COVID-19, non-COVID-19 viral pneumonia, bacterial pneumonia, and normal	2855	93.42%
[27]/2019	AlexNet, GoogLeNet and ResNet	Normal and Pneumonia	1431 pneumonia and 1431 normal	90%
[28]/2020	VGG-16	Normal, Bacterial Pneumonia and Virus Pneumonia	5232	93.0%
[29]/2021	InceptionResNetV2	Bacteria, Virus, normal, Pneumonia,	5232	90.7%
[30]/2021	Attention-based VGG-16	COVID, Normal, No_findings, Pneumonia Bacteria, Pneumonia Viral	Dataset 1–1125, Dataset 2–1638, Dataset 3–2138	79.58% 85.43% 87.49%
[31]/2021	Multi-scale bag of deep visual features with VGG	COVID, Normal, No_findings, Pneumonia Bacteria, Pneumonia Viral	Dataset 1–375, Dataset 2–1280, Dataset 3–1600, Dataset 4–276	84.37% 88.88% 90.30% 83.65%
[32]/2022	CNN + modified dropout Model	Healthy and Pneumonia	5856	91.0%
[33]/2022	Pre-activation ResNet with DenseNet169	Pneumonia and Non-Pneumonia	5856	90%
Proposed model	MobileNet	Pneumonia, Non-Pneumonia	Dataset 1- 5856, Dataset 2- 1,12,120	94.23% 93.75%

## Data Availability

Not applicable.
